# Starvation-Sensitized and Oxygenation-Promoted Tumor Sonodynamic Therapy by a Cascade Enzymatic Approach

**DOI:** 10.34133/2021/9769867

**Published:** 2021-06-02

**Authors:** Wencheng Wu, Yinying Pu, Han Lin, Heliang Yao, Jianlin Shi

**Affiliations:** ^1^The State Key Lab of High Performance Ceramics and Superfine Microstructures, Shanghai Institute of Ceramics, Chinese Academy of Sciences, Shanghai 200050, China; ^2^Center of Materials Science and Optoelectronics Engineering, University of Chinese Academy of Sciences, Beijing 100049, China; ^3^Department of Medical Ultrasound, Shanghai Tenth People's Hospital, Ultrasound Research and Education Institute, Tongji University School of Medicine, Shanghai 200072, China; ^4^Platform of Nanomedicine Translation, Shanghai Tenth People's Hospital of Tongji University, Shanghai 200072, China

## Abstract

The therapeutic outcomes of noninvasive sonodynamic therapy (SDT) are always compromised by tumor hypoxia, as well as inherent protective mechanisms of tumor. Herein, we report a simple cascade enzymatic approach of the concurrent glucose depletion and intratumoral oxygenation for starvation-sensitized and oxygenation-amplified sonodynamic therapy using a dual enzyme and sonosensitizer-loaded nanomedicine designated as GOD/CAT@ZPF-Lips. In particular, glucose oxidase- (GOD-) catalyzed glycolysis would cut off glucose supply within the tumor, resulting in the production of tumor hydrogen peroxide (H_2_O_2_) while causing tumor cells starvation. The generated H_2_O_2_ could subsequently be decomposed by catalase (CAT) to generate oxygen, which acts as reactants for the abundant singlet oxygen (^1^O_2_) production by loaded sonosensitizer hematoporphyrin monomethyl ether (HMME) upon the US irradiation, performing largely elevated therapeutic outcomes of SDT. In the meantime, the severe energy deprivation enabled by GOD-catalyzed glucose depletion would prevent tumor cells from executing protective mechanisms to defend themselves and make the tumor cells sensitized and succumbed to the cytotoxicity of ^1^O_2_. Eventually, GOD/CAT@ZPF-Lips demonstrate the excellent tumoral therapeutic effect of SDT in vivo without significant side effect through the cascade enzymatic starvation and oxygenation, and encouragingly, the tumor xenografts have been found completely eradicated in around 4 days by the intravenous injection of the nanomedicine without reoccurrence for as long as 20 days.

## 1. Introduction

Although great progress has been achieved on oncology over the decades, cancer remains one of the major threats to human health due to its high risk and mortality [1–3]. Given that traditional protocols (such as surgical excision, radiation therapy, and chemotherapy) would cause severe side effects and painful experiences on patients, noninvasive and safe sonodynamic therapy (SDT) shows promising prospects in cancer treatments [4–7]. In SDT, a combination of sonosensitizer(s), molecular oxygen (O_2_), and ultrasound could produce abundant singlet oxygen (^1^O_2_), which is a kind of reactive oxygen species (ROS) that can significantly induce cellular toxicity [8–10]. Even as one of the most promising noninvasive cancer treatment modalities, however, there are still two major obstacles that deteriorate the effectiveness of SDT in solid tumor therapy. Firstly, the yield amount of ^1^O_2_ is severely restricted by the hypoxia microenvironment of solid tumors [11–13]. On the other hand, the toxic effect of ^1^O_2_ on tumor cells would be compromised by their diverse intrinsic protective mechanisms [14, 15]. Hence, if the two major drawbacks can be overcome concurrently using a facile strategy, undoubtedly, the therapeutic effect of SDT can be greatly enhanced.

Fasting is a relatively gentle auxiliary therapeutic strategy, which can achieve short-term starvation by implementing a severely restricted diet [16]. Interestingly, the starvation caused by fasting has completely different effects on the metabolic activities of normal cells and tumor cells [17, 18]. Fasting-induced starvation would promote normal cells to redirect limited energy to the processes of cell maintenance and repair rather than growth or proliferation [19, 20]. Apparently, such an energy redistribution mechanism can protect normal cells from various cellular stresses (e.g., chemotoxicity) to a certain extent. On the contrary, under the promotion of oncogenes, the limited energy would be reallocated from tumor cell maintenance and repair to nutrient-deficient aberrant growth pathways, ultimately resulting in attenuated cellular stress tolerance [21, 22]. In this regard, short-term fasting might be applied as an effective intervention that can protect normal cells while enhancing the tumor cells-killing effect of SDT by suppressing various protective mechanisms of tumor cells. However, unlike normal lifestyle dietary regulation (that is, calorie restriction), fasting through severe food deprivation is undoubtedly painful and intolerable for most frail patients [16, 23]. Encouragingly, due to the unique Warburg effect of tumors caused by inefficient aerobic glycolysis making tumor cells more dependent on glucose supply than normal tissue cells, locally cutting off the glucose supply to tumor cells could rapidly and effectively achieve tumor starvation. The natural enzyme glucose oxidase (GOD), which can efficiently oxidize intracellular glucose to gluconic acid and H_2_O_2_ thereby cutting off the energy supply to cancer cells, is a highly preferred candidate for achieving tumor starvation [24, 25].

**Scheme 1:**
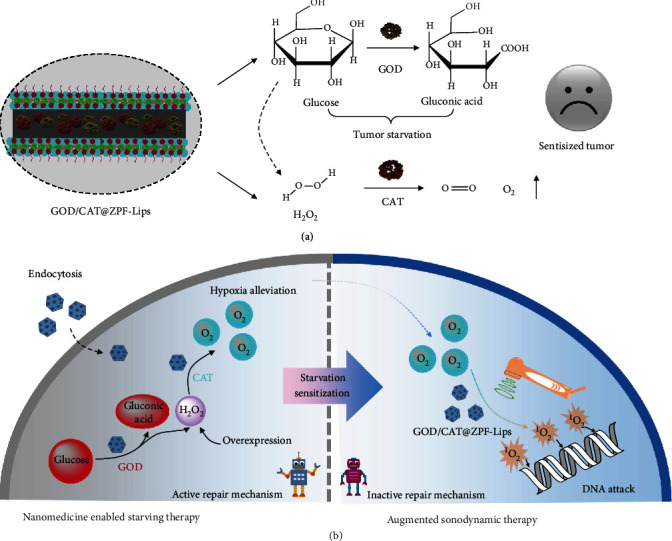
Schematic illustrations of liposome-modified nanomedicine-enabled glucose consumption and oxygen generation (a) and GOD/CAT@ZPF-Lips interaction with cancer cells enabling starvation/oxygenation-augmented SDT (b).

It is worth noting that the outcomes of both SDT treatment and GOD-mediated tumor starvation are heavily dependent on O_2_. Fortunately, H_2_O_2_ both produced by glucose metabolism and endogenously overexpressed in tumors can be immediately decomposed into O_2_ under the catalysis of another natural enzyme catalase (CAT). In turn, the generated O_2_ can not only promote the oxidation of glucose but also increase the production of cytotoxic ^1^O_2_ under the US irradiation through the SDT effect. Thus, the approach of introducing dual natural GOD and CAT enzymes into the tumor site for rapid glucose exhaustion and oxygen generation will be highly preferred for augmenting SDT. Herein, we design and synthesize a novel dual enzymes-encapsulated nanomedicine (GOD/CAT@ZPF) by immobilizing GOD and CAT into the frameworks of zeolitic pyrimidine, to concurrently trigger tumor starving and hypoxia relieving (Scheme 1(a)) and, subsequently, to augment SDT efficacy. To prolong their circulatory half-life for further application in vivo, GOD/CAT@ZPF nanoparticles were modified with liposomes embedded with a sonosensitizer hematoporphyrin monomethyl ether (GOD/CAT@ZPF-Lips). After the tumor accumulation of GOD/CAT@ZPF-Lips, glucose in tumor cells would be rapidly exhausted by encapsulated GOD causing localized and rapid glucose consumption and severely retarded glycolysis for adenosine triphosphate (ATP) production, finally leading to tumor starving. In the case of insufficient energy supply, tumor cells will more actively deprive the remaining energy to feed their abnormal growth rather than for cell maintenance and repair. Such a lopsided energy reallocation leads to the diminished ability of tumor cells to counteract cellular stress, including oxidative stress. Briefly, the enzymatic reaction of GOD concurrently depletes nutrient glucose (tumor starvation) and produces H_2_O_2_, and subsequently, CAT catalyzes the decomposition of H_2_O_2_ to generate O_2_ in series, resulting in starvation-sensitized tumor self-protective mechanism suppression and tumor hypoxia relieving, and consequently promoted ^1^O_2_ production upon the US irradiation (Scheme 1(b)). Finally, the SDT therapeutic effect on tumor cells is effectively augmented by starvation-enabled oxidative stress sensitizing and hypoxia relieving-promoted ROS production.

## 2. Results and Discussion

**Figure 1:**
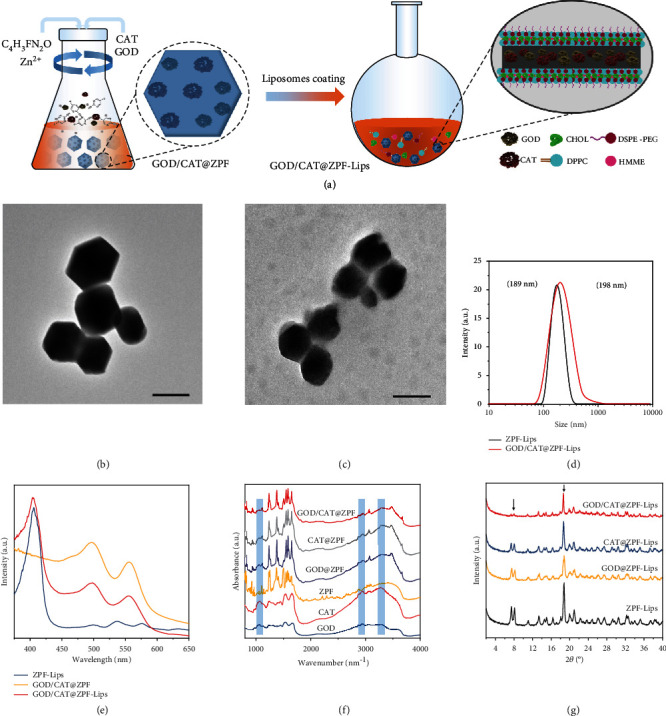
Fabrication and characterization of GOD/CAT@ZPF-Lips. (a) Schematics of GOD/CAT@ZPF-Lips fabrication. (b) TEM image of fresh ZPF-Lips, scale bar 200 nm. (c) TEM image of GOD/CAT@ZPF-Lips, scale bar 200 nm. (d) Hydrodynamic diameters of ZPF-Lips and GOD/CAT@ZPF-Lips in PBS as measured by DLS. (e) UV-Vis absorbance spectra of ZPF-Lips, GOD/CAT@ZPF, GOD/CAT@ZPF-Lips (where GOD and CAT are labeled with FITC and RB, respectively, and HMME is encapsulated into liposomes). (f) Fourier transform infrared (FTIR) spectra of different complexes. (g) XRD patterns of ZPF-Lips, GOD@ZPF-Lips, CAT@ZPF-Lips, and GOD/CAT@ZPF-Lips.

Initially, GOD and CAT were incorporated into the frameworks of zeolitic pyrimidine (GOD/CAT@ZPF) via in situ encapsulation method which is described in detail in the Supporting Information [26]. Subsequently, the biocompatibility of GOD/CAT@ZPF was improved by the decoration of HMME-embedded liposomes through a thin film hydration method, and the final products GOD/CAT@ZPF-Lips was obtained (Figure 1(a)) [27]. The prepared GOD/CAT@ZPF nanoparticles display a flat hexahedron morphology with a particle size of about 200 nm, and the decoration of liposomes did not significantly change their morphology and size as observed using transmission electron microscopy (TEM) (Figures 1(b) and 1(c)). Meanwhile, a thin film can be found on their surface, proving the successful modification of liposomes. This result is further confirmed by the changes of zeta potential of nanoparticles during the modification process, in which the surface charge of GOD/CAT@ZPF-Lips was measured to be -9.56 mV (Table S1). The hydrated particle size of GOD/CAT@ZPF-Lips measured by the dynamic light scattering (DLS) was slightly increased compared to the fresh ZPF-Lips due to the existence of GOD and CAT in ZPF (Figure 1(d)). To verify whether GOD and CAT were successfully immobilized in ZPF or not, we previously labeled the GOD and CAT with fluorescein isothiocyanate (FITC) and Rhodamine B (RB), respectively, for the subsequent synthesis. According to the UV-Vis absorption spectrum of GOD/CAT@ZPF (Figure 1(e)), there exist characteristic FITC and RB absorbances at 490 nm and 560 nm, respectively, indicating the successful immobilization of GOD and CAT, and the FTIR spectra further confirmed this result (Figure 1(f)). Additionally, the typical HMME absorbance at 410 nm was also detected in GOD/CAT@ZPF-Lips after modified with HMME-embedded liposomes, in which HMME acts as a sonosensitizer. Although the crystalline structure of ZPF was retained during the synthesis, but become deteriorated to a certain extent after encapsulating GOD and CAT as revealed by the XRD patterns (Figure 1(g)). Moreover, the encapsulation efficiency of GOD and CAT was calculated to be as high as 80% and 91%, respectively, which ensures the efficient enzymatic cascade reactions afterward.

**Figure 2:**
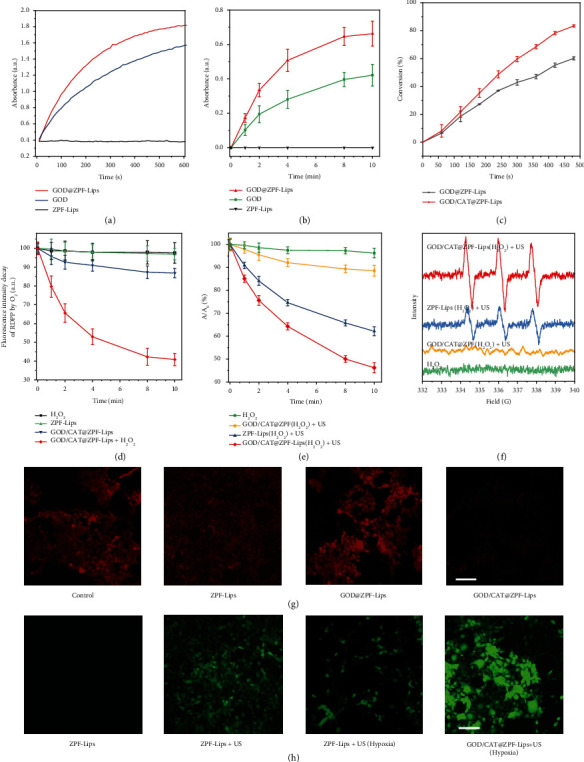
In vitro catalytic performance of GOD/CAT@ZPF-Lips. (a) Relative ABTS oxidation activities of ZPF-Lips, fresh GOD, and GOD@ZPF-Lips using a colorimetric assay based on a Cyt c-coupled system (*λ* = 660 nm). (b) UV-Vis spectra of H_2_O_2_ generated in the glucose solution (10 mM) after the reactions in the presences of ZPF-Lips, GOD, and GOD@ZPF-Lips at different time points (*n* = 3, independent experiments). (c) Catalytic activities of GOD@ZPF-Lips and GOD/CAT@ZPF-Lips based on the consumption kinetics of glucose. (d) The UV-Vis absorbances of RDPP in different reaction systems at varied time points (where the concentration of H_2_O_2_ is 1 × 10^−4^ M). (e) ROS-induced changes in the absorption of DPBF at 450 nm in different reaction systems, A0 is the initial absorbance of DPBF probe. (f) ESR spectra of different reaction systems showing the singlet oxygen generations under different treatments. (g) Fluorescence images of O_2_ generation in 4T1 cells in a hypoxic setting (i.e., N_2_ atmosphere) treated in different conditions, scale bar 100 *μ*m. (h) Confocal laser scanning microscopy (CLSM) images of 4T1 cells stained with SOSG after treating in different conditions in a 1 × 10^−4^ M H_2_O_2_ supplied setting, scale bar 100 *μ*m.

Considering that GOD and CAT have been encapsulated into ZPF capsules as expected, we comprehensively explored their biocatalytic activity in ZPF capsules in vitro, which is critical for promoting glucose consumption to achieve tumor cell starvation. We first performed a colorimetric assay based on the oxidation of ABTS by a Cyt c-coupled system to determine the catalytic activity of GOD in the ZPF. As evidenced by the enhanced chromogenic rate of GOD@ZPF-Lips, the activity of GOD@ZPF-Lips was increased compared to free GOD thanks to the full exposure of the active sites (Figure 2(a)). Additionally, the UV-Vis spectrophotometer was also applied to monitor the generation of hydrogen peroxide, one of the main products of GOD-mediated glucose metabolism, in different reaction systems over time. In the GOD@ZPF-Lips-mediated system, the absorbance increase of the generated H_2_O_2_ is significantly faster than that of the free GOD-mediated system, indicating the high enzymatic catalytic activity of GOD@ZPF-Lips (Figure 2(b)). In contrast, the characteristic absorption peak of H_2_O_2_ was not detected in the ZPF-Lips only system, which indicates that GOD plays an indispensable role in the GOD@ZPF-Lips nanomedicine. This result was further corroborated visually by the H_2_O_2_ fluorescent probe observation under a confocal microscope (Figure S1).

The cascade catalytic efficiency of GOD/CAT@ZPF-Lips was then assessed by detecting the consumption of glucose based on the 3,5-dinitrosalicylic acid (DNS) colorimetric assay. Comparatively, GOD/CAT@ZPF-Lips displayed an increased glucose consumption compared with GOD@ZPF-Lips, due to the fact that CAT catalyzed the production of O_2_ from H_2_O_2_ generated by glucose metabolism, further accelerating the enzymatic reaction of GOD (Figure 2(c)). To study the in vitro O_2_ generation kinetics of GOD/CAT@ZPF-Lips, we used [Ru(dpp)_3_]Cl_2_ (RDPP), whose fluorescence can be immediately quenched by O_2_, as an O_2_ probe to monitor O_2_ production. It can be found that H_2_O_2_ or free ZPF-Lips had no detectable effect on its fluorescence intensity, while GOD/CAT@ZPF-Lips caused a slight decrease in the fluorescence intensity of RDPP during the first few minutes, after which the fluorescence intensity loss remained unchanged in the following 5 min due to the self-consumption of O_2_ in the GOD/CAT cascade reaction (Figure 2(d)). However, after adding additional H_2_O_2_ (1 × 10^−4^ M) to the GOD/CAT@ZPF-Lips reaction system, a dramatic decrease of the fluorescence intensity of RDPP can be clearly observed, demonstrating that hypoxic conditions are effectively alleviated. Subsequently, the ability of GOD/CAT@ZPF-Lips to produce O_2_ in cells was also investigated, where RDPP was used as an indicator of intracellular O_2_ (red fluorescence is quenched by O_2_). For better simulating the tumor microenvironment (TME) containing endogenous H_2_O_2_, the culture was supplemented with 1 × 10^−4^ M H_2_O_2_. In all groups, cells treated with GOD/CAT@ZPF-Lips exhibited the lowest fluorescence even cultured in the N_2_ atmosphere, demonstrating that GOD/CAT@ZPF-Lips are capable of alleviating intracellular hypoxia by generating O_2_ (Figure 2(g)).

With the sufficient oxygen supply, HMME in GOD/CAT@ZPF-Lips can generate abundant toxic ^1^O_2_ upon the US irradiation for achieving effective SDT treatment. Hence, ^1^O_2_ produced by GOD/CAT@ZPF-Lips was analyzed by a 3-diphenylisobenzofuran (DPBF) assay and electron spin resonance (ESR) spectroscopy. As a ^1^O_2_ probe, DPBF will be irreversibly oxidized by the ROS, resulting in a decrease in its characteristic absorbance at 410 nm. As shown in Figure 2(e), GOD/CAT@ZPF combined with either the US irradiation or H_2_O_2_ only, induced a negligible decrease in the absorption intensity of DPBF at 410 nm, while a rapid decrease occurred after combining HMME-Lips and the US irradiation, which manifests that the HMME loaded in the liposome is responsible for the generation of ^1^O_2_ instead of GOD/CAT@ZPF itself. Notably, the decrease of the absorption intensity of DPBF at 410 nm was further intensified under the combined GOD/CAT@ZPF-Lips and the US irradiation, indicating more amount of ^1^O_2_ had been generated owing to the additional oxygen supplementation from GOD/CAT cascade reaction. Additionally, by using 2, 2, 6, 6-tetramethyl 1-4 piperidone hydrochloride (TEMP) as a spin trap for ^1^O_2_, ESR quantitatively proved the effective production of ^1^O_2_. Compared to the control groups (H_2_O_2_ only or GOD/CAT@ZPF+US), significant characteristic ^1^O_2_ 1 : 1 : 1 signals were detected in the group of ZPF-Lips+US and GOD/CAT@ZPF-Lips. And after introducing GOD/CAT cascade reaction with additional H_2_O_2_, the signal of ^1^O_2_ radicals in the GOD/CAT@ZPF-Lips+US group was significantly augmented, which is consistent with DBPF measurements (Figure 2(f)).

Following the acellular solution-based tests, the capability of GOD/CAT@ZPF-Lips to intracellularly generate ^1^O_2_ was further evaluated. The prerequisite for intracellular generation of ^1^O_2_ is the efficient cellular uptake of GOD/CAT@ZPF-Lips. To visually observe the internalization of GOD/CAT@ZPF-Lips by cancer cells, we prepared FITC-labeled GOD and RB-labeled CAT for monitoring their behavior in cells. After the incubation of mice, malignant breast cancer cells (4T1) with FITC and RB-labeled GOD/CAT@ZPF-Lips for 0.5 h, both green FITC and red RB fluorescence were observed under CLSM, demonstrating the effective accumulation of GOD/CAT@ZPF-Lips in cells by endocytosis. Meanwhile, the complete overlap between red and green fluorescences indicates once again that both GOD and CAT have been successfully immobilized in the ZPF nanoparticles (Figure S2). Next, we used single oxygen sensor green (SOSG), a ^1^O_2_ specific fluorescent probe that can react with ^1^O_2_ and then generate a green fluorescence endoperoxide, to monitor intracellular ^1^O_2_ levels in different groups. It can be seen in Figure 2(h) that the ZPF-Lips treatment group did not exhibit significant green fluorescence. In the ZPF-Lips+US group, although considerable green fluorescence was observed at first, which was then significantly decreased once the 4T1 cells were located in a hypoxic environment, suggesting that sufficient O_2_ is a prerequisite for abundant ^1^O_2_ production. Of note, in the same hypoxic culture environment, the bright green fluorescence still could be observed in GOD/CAT@ZPF-Lips+US group, which can be attributed to the efficient tumor cells hypoxia remission by GOD/CAT@ZPF-Lips. These data demonstrate that the designed nanomedicine is competent to produce a significant amount of O_2_ in tumor cells for the subsequent efficient ^1^O_2_ generation upon the US irradiation.

**Figure 3:**
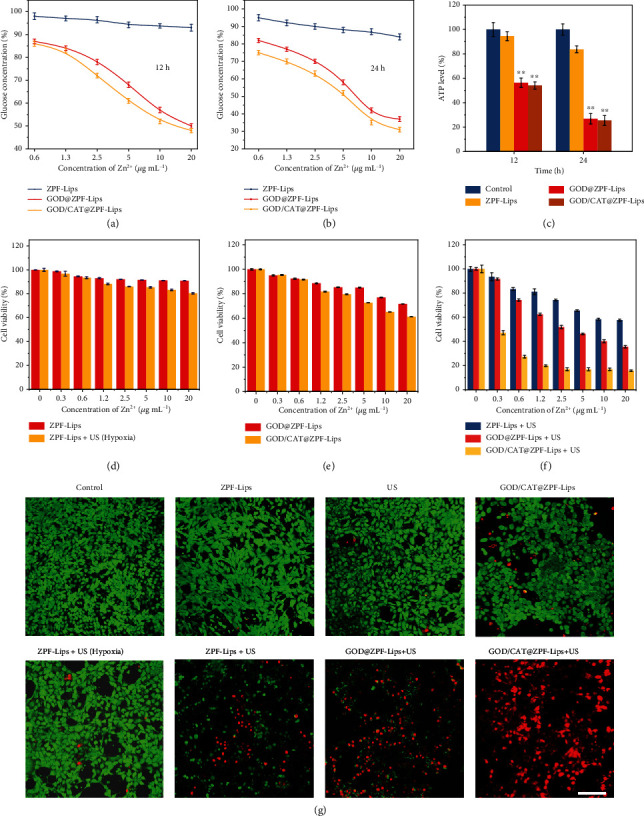
In vitro evaluations of glucose consumption and cytotoxicity of GOD/CAT@ZPF-Lips. (a, b) Extracellular glucose concentrations of 4T1 cells after various treatments for 12 and 24 h. (c) Intracellular ATP levels in 4T1 cells after various treatments for 12 and 24 h. (d–f) In vitro viability assays of 4T1 cells after various treatments for 24 h. (g) CLSM images of 4T1 cells after treated in different conditions and subsequently stained by Calcein-AM/PI (scale bar: 100 *μ*m).

As stated above, GOD is an enzyme that can catalyze the rapid consumption of intracellular glucose leading to the insufficient main energy supply for cancer cells. This event subsequently induces the decrease of ATP production from glycolysis, severely depriving the energy for cancer cell proliferation. Accordingly, we can rationally expect that GOD-enabled tumor starvation may enhance US-triggered sonodynamic therapy for further promoted therapeutic efficacy. To this end, we explored the effects of ZPF-Lips, GOD@ZPF-Lips, and GOD/CAT@ZPF-Lips on glycolysis at the cellular level. 4T1 cells were incubated with different concentrations of ZPF-Lips, GOD@ZPF-Lips, and GOD/CAT@ZPF-Lips for 12 h or 24 h. As a substrate of glycolysis, glucose in 4T1 cells was significantly decreased after GOD@ZPF-Lips and GOD/CAT@ZPF-Lips treatments (Figures 3(a) and 3(b)). Either elevating GOD concentrations or prolonging GOD incubation durations could further aggravate the consumption of intracellular glucose (maximum 69.4% consumption). In contrast, ZPF-Lips alone failed to induce comparable glucose depletion, though the high equivalent Zn^2+^ concentration (20 *μ*g mL^−1^) from ZPF-Lips did initiate the consumption of glucose (16.3%) in 4T1 cells probably due to the normal stimulating metabolism effects of ZPF-Lips. Such a disordered glucose depletion would result in the insufficient production of ATP, the main energy in malignant cells, thereby failing to meet their large energy demands. To determine the ATP level in differently treated cells, the ATP detection kit was applied. As illustrated in Figure 3(c), ATP production in 4T1 cells was significantly reduced after GOD@ZPF-Lips or GOD/CAT@ZPF-Lips treatment, especially in 24 h of incubation. In free ZPF-Lips groups, however, no obvious changes in ATP production were detected, indicating that ZPF-Lips alone cannot affect energy metabolism.

For comprehensively investigating whether GOD-enabled tumor cell starvation could potentiate the killing effect of GOD/CAT@ZPF-Lips on tumor cells after the US irradiation or not, a standard Cell Counting Kit-8 (CCK-8) assay was applied to assess the antineoplastic effect of different treatments. As shown in Figure 3(d), ZPF-Lips alone had a minor influence on the viabilities of 4T1 cells. And under hypoxic conditions, ZPF-Lips only killed a small percentage of cells despite the combination of the US irradiation due to the limited production of ^1^O_2_ by hypoxia. While GOD@ZPF-Lips showed certain toxicity to 4T1 cells owing to the consumption of glucose and the accumulation of gluconic acid in the cells. Of note, the reduction in cell viability is slightly increased after incubated with GOD/CAT@ZPF-Lips at the same GOD@ZPF-Lips concentration, which is attributed to the promotions of the GOD-mediated glucose oxidation by CAT (Figure 3(e)). Compared with the less strong cytotoxicity under hypoxic conditions, ZPF-Lips+US exhibited significantly enhanced and concentration-dependent toxicity to tumor cells in a normal culture atmosphere (80.4% *vs.* 61.2% when [Zn^2+^] = 20 *μ*g mL^−1^, Figure 3(f)); nevertheless, in this case, quite a few cancer cells still survived via the intrinsic self-repair function to counteract the attack by limited amount of ^1^O_2_ generated by US. While cells in a starving and hypoxic state induced by GOD@ZPF-Lips will be forced to downregulate various metabolic and self-repair functions to counteract the lack of energy supply. As a result, after treated by GOD@ZPF-Lips, the starving cells are less capable of resisting the attacks of ^1^O_2_ when exposed to the US irradiation, ultimately obtaining effective anticancer outcomes in which most 4T1 cells have succumbed to ^1^O_2_-induced oxidative damage.

More importantly, CAT is able to decompose intratumoral H_2_O_2_ into O_2_, which can provide enough reactants for the HMME-mediated sonocatalytic reaction to generate abundant ^1^O_2_, further elevating the cell-killing efficacy of GOD/CAT@ZPF-Lips. (Figure 3(f)). Additionally, such a synergistic antitumor effect of GOD/CAT@ZPF-Lips+US was further confirmed by a calcein acetoxymethyl ester (Calcein-AM)/PI (propidium iodide) staining assay, where live and dead cells were visualized by green and red fluorescences, respectively (Figure 3(g)). Intensive green Calcein-AM fluorescence was observed in the control, ZPF-Lips, and US groups, which indicates that those treatments will no induce significant cell toxicity. In addition, neither SDT alone (ZPF-Lips+US (hypoxia)) nor starving therapy alone (GOD/CAT@ZPF-Lips) demonstrate sufficient cancer cell killing effect, as indicated by the low red PI signals in these two groups. Although red fluorescence could be clearly observed in ZPF-Lips+US group, relatively strong green fluorescence is also present, revealing that a large number of cancer cells have survived from the attack by ^1^O_2_. The increased red PI signals after GOD@ZPF-Lips+US treatment suggests that GOD-induced starvation therapy has sensitized cancer cells to be vulnerable to the ^1^O_2_-mediated cytotoxicity, leading to intensified cell death. As expected, the synergistic therapeutic effect of starving therapy and SDT could be further elevated by enhanced amounts of O_2_ and ^1^O_2_ via CAT-catalyzed H_2_O_2_ decomposition and the sonosensitizer-enabled O_2_ transition to ^1^O_2_, respectively, as determined by the bright red PI fluorescence in the GOD/CAT@ZPF-Lips+US treatment group. These results indicate that both GOD and CAT play key roles in the ^1^O_2_ production under the presence of sonosensitizer HMME and the US irradiation.

**Figure 4:**
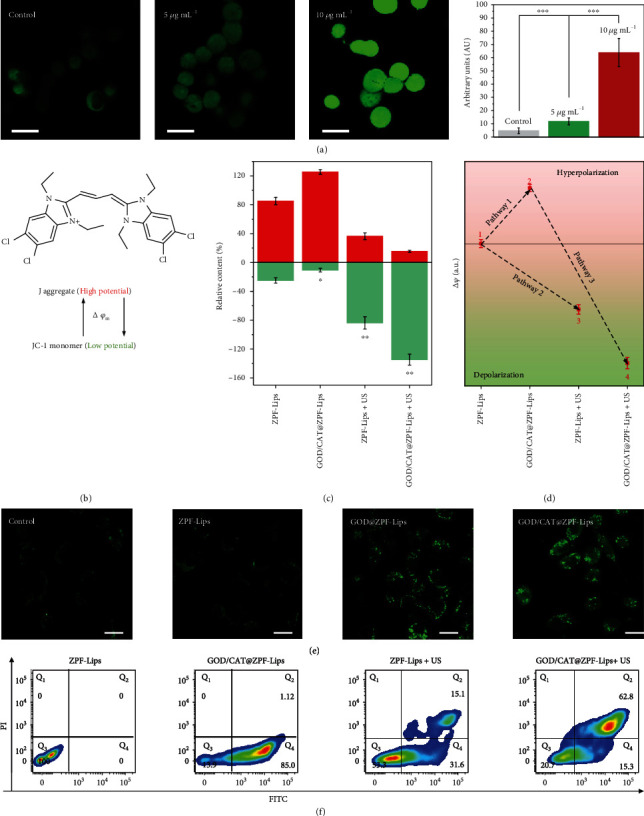
(a) CLSM images of 4T1 cells stained by glucose uptake and transport probe 2-NBDGT1, and the corresponding mean fluorescence intensities (scale bar: 20 *μ*m) upon treated with different amounts of GOD@ZPF-Lips. (b) Schematic illustration of the reversible conversion of JC-1 monomer into J-aggregate accompanied by increased *ΔΨ*m. (c) Relative amounts of J-aggregate and JC-1 monomer in the mitochondria of 4T1 cells after different treatments. (d) Hyperpolarization/depolarization of mitochondria in cancer cells as indicated by the semiquantitative determination of *ΔΨ*m. (e) CLSM images of 4T1 cells after treated in different conditions for 8 h and subsequently stained with cellular senescence probe SPiDER-*β*Gal (scale bar: 20 *μ*m). Data are expressed as means ± SD (*n* = 6). (f) Flow cytometry analyses for characterizing the apoptosis of Annexin V-FITC/PI-stained 4T1 cells treated under different conditions.

Next, we applied 2-NBDGT1 as a fluorescence probe to monitor the glucose uptake and transport behaviors of cells after treated with different concentrations of GOD@ZPF-Lips for 8 h. It can be noticed that the cellular glucose uptake and transport activity of the experimental groups are stronger than that of the control group and concentration-dependent. Such a strong cellular demand for glucose proves that the tumor cells are in a starved state after the treatment with GOD@ZPF-Lips (Figure 4(a)). Mitochondria, as power pumping stations for abundant energy production in cells, require a continuous intake of “fuel” such as glucose [28], so we assume that GOD-mediated glucose depletion may have an intensified impact on the mitochondrial function. Specifically, mitochondrial dysfunction will induce increased mitochondrial outer membrane permeability and subsequent cytoplasmic release of proapoptotic proteins, accelerating cell death [29].

To validate this assumption, we investigated the mitochondrial transmembrane potential (*ΔΨ*_m_) change of 4T1 cells after undergoing different treatments, which is a typical feature of mitochondria that reveals their hyperpolarization or depolarization. Herein, 5,5',6,6'-tetrachloro-1,1',3,3'-tetraethylbenzimidazolocarbocyanine iodide (JC-1) [30, 31], a J-aggregate-forming delocalized lipophilic cation, was used as a potentiometric probe to offer semiquantitative information on *ΔΨ_m_* (Figure 4(b)). J-aggregate amount in 4T1 cells increased dramatically after the GOD/CAT@ZPF-Lips treatment (Figure 4(c)), indicating that glucose depletion-induced starvation has led to mitochondrial hyperpolarization (Pathway 1 in Figure 4(d)). Conversely, the Jaggregate amount decreased in 4T1 cells after ZPF-Lips+US treatment, demonstrating that conventional SDT has triggered depolarization of mitochondria favoring apoptosis (Pathway 2). More interestingly, this effect became much more pronounced in the GOD/CAT@ZPF-Lips+US group. Herein, it can be proposed that in this scenario, the former hyperpolarization triggered by GOD (namely, sensitization state) has enabled the latter depolarization by ^1^O_2_ generated during SDT (activation state), finally resulting in a more remarkable mitochondrial permeability transition (Pathway 3).

To further uncover the mechanism underlying the starvation sensitization effect of GOD/CAT@ZPF-Lips, we explored the cellular pathway that guides cancer cell death. The expression of *β*-galactosidase (SA-*β*-gal), a typical signal of senescence, was detected in 4T1 cells by SPiDER-*β*Gal after treatments under different conditions. As a consequence, GOD/CAT@ZPF-Lips induced the highest expression of SA-*β*-gal in cells, evidencing that the GOD-enabled tumor starvation would exacerbate cell senescence, which may promote tumor cell apoptosis when encountering additional lethal attacks. The pathways of cell death in different groups were also addressed in detail by flow cytometry by FITC-labeled Annexin V and PI stainings (Figure 4(f)). The mitochondrial dysfunction induced by GOD/CAT@ZPF-Lips-enabled starvation has mainly caused considerable early apoptosis of 4T1 cells (Q_4_ quadrants), which is regarded to be not lethal due to the absence of SDT. In the ZPF-Lips+US group, 4T1 cells present relatively strong early apoptosis but less significant late apoptosis (Q_2_ quadrants), demonstrating the less therapeutic effectiveness of SDT alone. However, a sharp increase in the amount of late apoptotic cells was detected in GOD/CAT@ZPF-Lips+US group. These synergistic therapeutic outcomes confirm that the starvation-induced and SDT-enabled mitochondrial proapoptotic pathway is responsible for the significantly elevated tumor cell-killing efficacy by the GOD/CAT@ZPF-Lips+US treatment.

**Figure 5:**
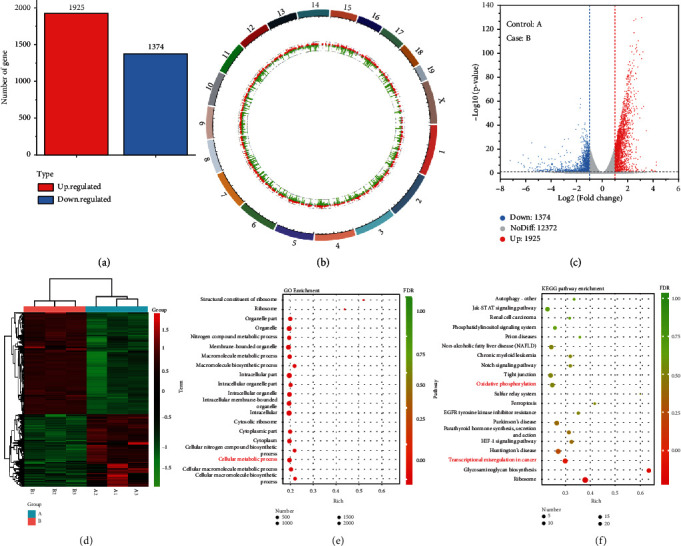
Transcriptomic analysis. In the figure, A and B represent the control and the GOD/CAT@ZPF-Lips group, respectively. (a) Statistics of the differentiated genes between group A and group B. (b) Genome circle map and (c) the volcano plot showing the distribution of genes and the results of significant differences in genes after treated by GOD/CAT@ZPF-Lips. (d) The heat map representation of differentially expressed genes which altered mRNA transcripts in 4T1 cells. (e) GO and (f) KEGG enrichment analysis for studying the underlying pathways of GOD/CAT@ZPF-Lips treatment.

We also performed mRNA sequencing on free DMEM- and GOD/CAT@ZPF-Lips-treated 4T1 cells to study their gene expression changes for better understanding its underlying biomechanism. A total of 15671 genes of 4T1 cells were analyzed in this assay, in which those of transcription fold change value >2 and *P* value < 0.05 were designated as differentially expressed genes. It has been found that there are 3299 differentially expressed genes between control and GOD/CAT@ZPF-Lips treatment groups, including 1374 downregulated and 1925 upregulated genes (Figures 5(a)–5(c)). In addition, the genes that consistently and significantly changed were compiled and visualized in the form of a heat map (Figure 5(d)). The significant dysregulation of genes in GOD/CAT@ZPF-Lips-treated 4T1 cells indicates that starvation induced by glucose depletion has an appreciable impact on its transcriptional behavior, thereby leading to other dysfunctions. To reduce the complexity of obtained differentially expressed genes, Gene Ontology (GO) functional annotations analysis was performed, in which all expressed genes were classified into Molecular Function (MF), Cellular Component (CC), and Biological Process (BP) (Figure S3). The 20 top significant enrichment GO terms are shown in Figure 5(e). Of note, GO terms associated with cellular metabolism are included in the top 20 significant enrichment GO terms of the biological process, which indicates that GOD/CAT@ZPF-Lips has significantly influenced the metabolic functions of 4T1 cells in GOD/CAT@ZPF-Lips/4T1 cells coculture model. After that, the Kyoto encyclopedia of genes and genomes (KEGG) enrichment analysis was used to further explore the signaling pathways involved in the direct stimulation of 4T1 by GOD/CAT@ZPF-Lips. A total of 296 remarkable enriched pathways were obtained after carrying out KEGG signal pathway analysis of different genes. Then, we focused on the top 20 out of 296 significant enriched pathways, of which two pathways were further investigated (Figure 5(f)). The one is “oxidative phosphorylation” linked to “energy metabolism” and the other one is “transcriptional misregulation in cancer” and relevant to “signal transduction” (Figure S4). In view of this, GOD/CAT@ZPF-Lips is believed to disrupt the cellular metabolism by changing their gene expressions, promoting the apoptosis of 4T1 cells once attacked by ^1^O_2_.

**Figure 6:**
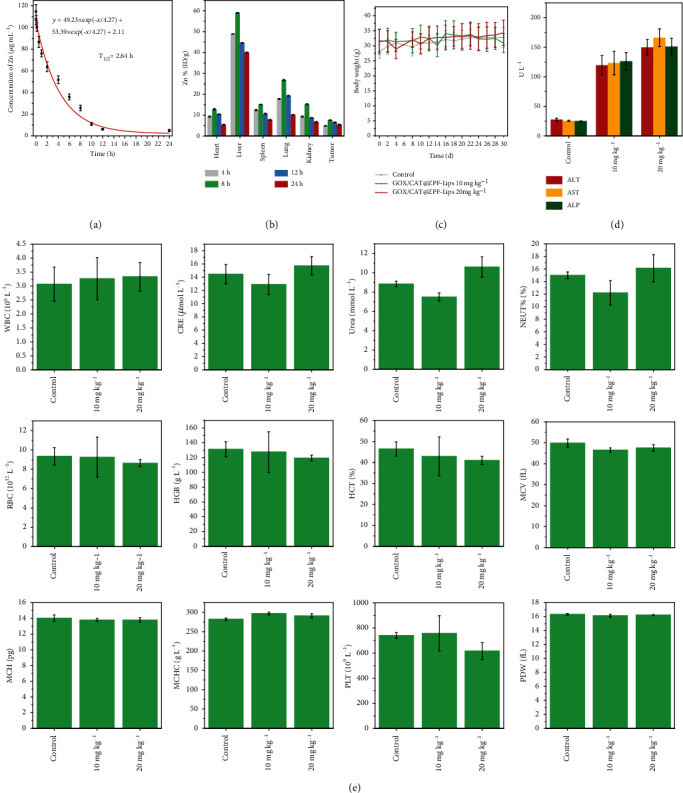
In vivo biological behavior of GOD/CAT@ZPF-Lips. (a) Blood-circulation lifetime of GOD/CAT@ZPF-Lips in mice after intravenous administration via tail veins (*n* = 3). (b) Biodistribution of Zn^2+^ after injecting GOD/CAT@ZPF-Lips (20 mg kg^−1^) into the mice for 4, 8, 12, and 24 h. (c) Body weights of Kunming mice treated under different conditions during 30 days of feeding. (e, d) Hematological assays of Kunming mice in 30 days posttreatment in different conditions.

Before being applied for in vivo anticancer therapy, the in vivo biological behavior of GOD/CAT@ZPF-Lips, including blood-circulation, biodistribution, and biocompatibility, was systematically assessed. Based on the two-compartment model, the blood-circulation half-time of the carrier ZPF-Lips was calculated to be as long as 2.64 h, benefiting from the protection of outer liposomes (Figure 6(a)). Thanks to the long blood-circulation time duration, GOD/CAT@ZPF-Lips could effectively accumulate inside the tumor through the enhanced permeability and retention (EPR) effect during circulation. As the main metal element of ZPF-Lips, the biodistribution result of Zn demonstrates that ZPF-Lips is capable of accumulating and remaining inside tumor for a relatively long duration, from which the tumor passive-targeting efficiency has been calculated to be 7.65% in 8 h postinjection. Moreover, an imaging assay was performed by injecting IR783-labelled ZPF-Lips into tumor-bearing mice with different tumor size (20 mg kg^−1^,100 *μ*L) for tracking theirs in vivo behaviors. It was observed that in all mice the fluorescence intensity at the tumor site gradually increased within 8 h, while the fluorescence intensity in other major organs of mice began to gradually decrease in 2 h, elucidating that ZPF-Lips possess effective EPR-derived tumor accumulation and excellent in vivo degradability (Figure S5). Subsequently, to systematically investigate the biocompatibility of GOD/CAT@ZPF-Lips without the US irradiation, healthy Kunming mice were randomly divided into 3 groups (*n* = 5) and injected intravenously with PBS and GOD/CAT@ZPF-Lips with different doses (10, 20 mg kg^−1^), respectively. No significant weight loss was observed in all groups of mice during the 30 days feeding duration (Figure 6(c)). Moreover, the mice were sacrificed at the end of observations to collect their blood and major organs (heart, liver, spleen, lung, and kidney) for further systematic evaluation. The hepatic function indexes (ALT, AST, and ALP) of mice in all groups did not show significant differences, which demonstrate that the adverse effect exerted by GOD/CAT@ZPF-Lips on liver functions are negligible. Besides, there were no obvious abnormalities in other important blood indexes of mice in the two experimental groups compared with the control group (Figure 6(e)). In addition to blood indexes evaluations of mice, hematoxylin and eosin (H&E) staining of major organs (heart, liver, spleen, lung, and kidney) in different groups also showed negligible acute, chronic adverse effects, further evidencing the biocompatibility of the designed GOD/CAT@ZPF-Lips in vivo (Figure S6).

**Figure 7:**
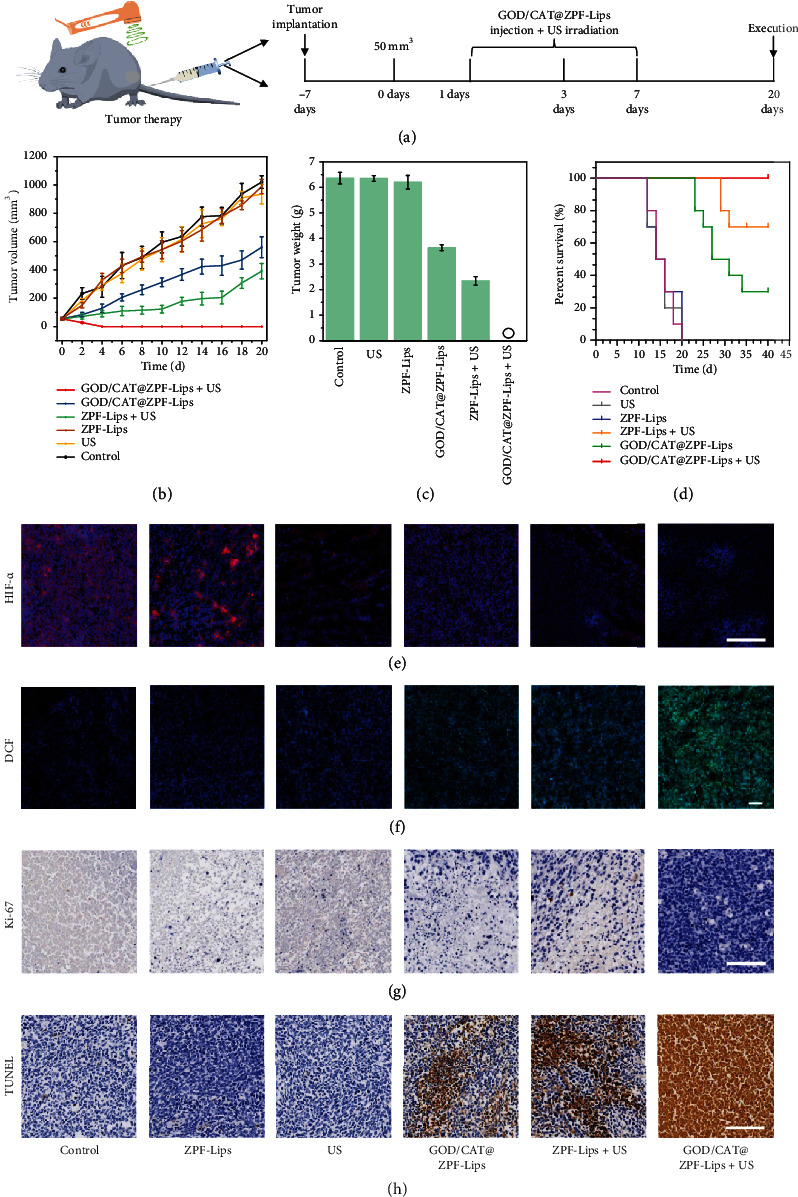
In vivo tumor therapeutic effect of Cu-LDH/HMME@Lips on the 4T1 tumor-bearing mice. (a) Schematics of the establishment of 4T1 tumor-bearing mouse model and in vivo treatment process via tail vein injection at the same ZPF dose of 20 mg kg-1. (b) Time-dependent tumor volume change curves after various treatments and (c) the final tumor weight of mice in different groups. (d) Kaplan-Meier survival curves of 4T1 tumor-bearing mice in the different groups, during the whole 20 days of observation, no tumor reoccurrence has been observed in the mice of GOD/CAT@ZPF-Lips+US group. (e) Ex vivo immunofluorescence images of tumor sections, in which the nucleus and hypoxic regions were stained by DAPI (blue) and HIF-*α* antibody (green), respectively. (f) ROS fluorescence images of tumors after various treatments for 12 h. (g, h) Ki-67 immunofluorescence labeling and TUNEL staining of 4T1 tumor xenograft sections in 12 h posttreatments (scale bar: 100 *μ*m). Scale bars in all groups are 100 *μ*m.

The excellent cancer cell-killing efficacy in vitro, great biological behavior, as well as biocompatibility in vivo stimulated us to investigate the therapeutic potential of GOD/CAT@ZPF-Lips in vivo on 4T1 subcutaneous tumor-bearing mice. The 4T1 xenograft tumor model was established by subcutaneously injecting 4T1 cells into the right hind leg of four-week-old female Balb/c nude mice. When the tumor size reached around 50 mm^3^, these 4T1 tumor-bearing nude mice were randomly divided into six groups: PBS (control), US, ZPF-Lips, ZPF-Lips+US, GOD/CAT@ZPF-Lips, and GOD/CAT@ZPF-Lips+US (Figure 7(a)). During an observation period of 20 days, the body weight and tumor volumes were recorded every other day. As Figures 7(b) and 7(c) illustrated, both the ZPF-Lips+US and GOD/CAT@ZPF-Lips treatments show significant but moderate tumor growth inhibition effects compared with the other three treatments (PBS, US, and ZPF-Lips), confirming that the effectiveness of neither SDT nor starving therapy alone is satisfactory. Notably and importantly, the tumors of mice in the GOD/CAT@ZPF-Lips+US group were eliminated on the fourth day of treatment, manifesting to the excellent anticancer efficacy (~100% tumor removal) of the cascade enzamic-promoted and starvation-sensitized SDT (Figure S7a).

More detailed tumor hypoxic and oxygenation behaviors by the nanomedicine were examined. It can be seen that the hypoxia environment in solid 4T1 tumors is effectively relieved by O2 generation from CAT-enabled H2O2 decomposition (Figure 7(e)). Therefore, more toxic ^1^O_2_ would be produced once exposed to the US irradiation, achieving an elevated HMME-mediated SDT therapeutic efficacy (Figure 7(f)). On the other hand, the GOD-induced starving effect sensitized tumor cells to the attacks by, e.g., ^1^O_2_, thereby leading to pronounced tumor cell killing by ^1^O_2_ cytotoxicity upon the US irradiation. Such significant synergistic therapeutic outcomes prove that the starvation-sensitization by natural GOD is an efficient route to potentiate SDT for clinic translation. Further, mice in PBS (control), US, ZPF-Lips, ZPF-Lips+US, and GOD/CAT@ZPF-Lips groups gradually died due to the aggressive invasions of tumors. However, all of the mice in the GOD/CAT@ZPF-Lips+US group survived over a period of as long as 20 days without visible tumor reoccurrence thanks to the high therapeutic efficacy of the synergistic therapy (Figure 7(d)). Neither significant body weight fluctuations of mice in all groups during the evaluation period, nor visible pathological changes in the main organs of mice stained with the hematoxylin and eosin (H&E) after the treatments, were found (Figure S7b, Figure S8). These results indicate the negligible harmful side effects of these treatments on the mice. We further conducted the immunohistochemical assays of Ki-67 and terminal deoxynucleotidyl transferase dUTP nick end labeling (TUNEL) for xenografted tumor sections to further investigate the therapeutic mechanism of GOD/CAT@ZPF-Lips with the US irradiation. Both ZPF-Lips+US and GOD/CAT@ZPF-Lips induce considerable downregulations of the Ki-67 level compared to the other three treatments (PBS, US, and ZPF-Lips), verifying the inhibitory effects of both SDT and starvation therapy on cancer cell proliferation. Not surprisingly, the inhibition effect of cancer cell proliferation was further augmented by synergistic therapy (GOD/CAT@ZPF-Lips+US) (Figure 7(g)). Similarly, TUNEL immunofluorescent assays of tumor sections also validate this conclusion, in which the treatment of GOD/CAT@ZPF-Lips+US results in maximum tumor cell apoptosis (Figure 7(h)).

## 3. Conclusion

In this work, we have constructed a novel nanomedicine with dual enzyme activities, designated as GOD/CAT@ZPF-Lips, for in situ triggering tumor starvation and oxygenation concurrently to amplify SDT. Cutting off glucose supply by GOD predisposes cancer cells to severe energy deficiency and subsequently induces tumor starvation, and such starving tumor cells are unable to obtain enough energy for cell maintenance and repair, making tumor cells highly sensitive to oxidative stress. Attractively, the by-product, H_2_O_2_ during the glucose oxidation by GOD, provides sufficient reactant for the O_2_ generation under the catalysis of CAT and, more importantly, relieves tumor hypoxia for promoting SDT-mediated ^1^O_2_ generation. As a result, starving tumor cells become sensitized and completely succumbed to the cytotoxicity of ^1^O_2_ produced via the dual enzymatic reactions. Both in vitro and in vivo results prove the sensitizing effect of starvation for tumor cells, which leads to a significant superadditive therapeutic outcome without significant side effect, and even tumor eradication by intravenous injection without reoccurrence in the following 20 days. It is expected that such a combined cascade enzymatic strategy of concurrently cutting off endogenous nutrition supply and sensitizing tumor cell to oxidative stress, and consequently promoting intratumoral oxygenation may be highly informative for the future design of nanomedicine to elevate SDT.

## 4. Materials and Methods

### 4.1. Chemicals and Reagents

Zinc nitrate hexahydrate and hydrogen peroxide (H_2_O_2_) were obtained from Adamas-Beta Co. (Shanghai, China). 2-chloro-5-fluoropyrimidine was obtained from Adamas (Shanghai, China). 3,5-dinitrosalicylic acid (DNS) solution was purchased from Dingguo (Beijing, China). Diammonium 2,2'-azino-bis(3-ethylbenzothiazoline-6-sulfonate) (ABTS), Catalase (CAT) was purchased from Sigma-Aldrich (Shanghai, China). Glucose oxidase (GOD) and HEPES buffer (2.57 mL, 50 mM, pH = 7) were purchased from Solarbio (Beijing, China). Hematoporphyrin monomethyl ether (HMME), 1,3-diphenylisobenzofuran 1,3 (DPBF), Dimethyl sulfoxide (DMSO), singlet oxygen sensor green (SOSG), and [Ru(dpp)_3_]Cl_2_ (RDPP) were purchased from Sigma-Aldrich Co. (Shanghai, China). Dulbecco's modified eagle medium (DMEM high glucose), calcein, 4, 6-diamidino-2-phenylindole (DAPI), propidium iodide (PI), and cell counting Kit-8 (CCK-8) were ordered from United Bioresearch, Inc. 2-NBDG, fluorescein isothiocyanate (FITC), cellular senescence detection kit-SPiDER-*β*Gal, and rhodamine B were purchased from Beyotime Biotechnology Co. (Haimen, China).

### 4.2. Fabrication of Fresh GOD/CAT@ZPF Nanoparticles

GOD/CAT@ZPF was synthesized by the one-pot method. In detail: zinc nitrate aqueous solution (2 mL59.7 mg mL^−1^) was added into to a DI water solution (4 mL) containing 2-hydroxy-5-fluoropyrimidine (138.4 mg), GOD (5.0 mg), and CAT (10 mg). The reaction mixture was stirred for 20 minutes at 25°C. The product was centrifugated and washed with DI water for three times to remove the residual impurity. For the preparation of GOD@ZPF or CAT@ZPF, the same protocol was applied where either GOD (12 mg) or CAT (12 mg) was added instead of the mixed GOD&CAT (5.0 mg, 10 mg).

### 4.3. Characterization

The hydrodynamic size distribution of nanoparticles was determined by a Malvern Zetasizer Nano series (Malvern Panalytical, Malvern, UK). X-ray diffraction measurements (XRD Bruker D8 Focus, Bruker, Billerica, MA, USA; 2*θ* ranging from 0° to 40° Cu K_*α*1_) were performed on free ZPF, CAT@ZPF-Lips, GOD@ZPF-Lips, and GOD/CAT@ZPF-Lips powder. Their morphology was obtained by a transmission electron microscopy (JEM-2100F, Tokyo, Japan) which was working at an accelerating voltage of 200 kV. Electron spin resonance (ESR) measurements were performed on a jeol-fa200 spectrometer at room temperature with the following settings: microwave frequency = 9.425 GHz, microwave power = 0.998 mW, modulation frequency = 100.00 kHz, and modulation amplitude = 2.00 G. TEMP were used as a spin trap of ^1^O_2_. The encapsulation efficiency of HMME in the liposomes and the immobilization efficiency of GOD and CAT in ZPF were measured by UV-Vis spectra technology (UV-3600 Shimadzu).

### 4.4. Enzymatic Activity of GOD

The colorimetric assay based on the oxidation of Diammonium 2, 2'-azino-bis(3-ethylbenzothiazoline-6-sulfonate) (ABTS) by a Cyt c-coupled system (*λ* = 660 nm) was applied to explore GOD activity. The activity of GOD was determined through a colorimetric assay based on the oxidation of Diammonium 2, 2'-azino-bis(3-ethylbenzothiazoline-6-sulfonate) (ABTS) by a Cyt c-coupled system (*λ* = 660 nm). Typically, suspension solution of ZPF, GOD@ZPF-Lips (Zn^2+^: 20 *μ*g mL^−1^), or free GOD (100 *μ*L) was added into solution containing glucose (100 *μ*L, 200 mM) in HEPES buffer (2.57 mL, 50 mM, pH = 7.4), the mixtures were placed at RT for 15 min. Then, ABTS (300 *μ*L, 5 mM) and Cyt c (30 *μ*L, 1 mg mL-1) were added. Immediately, in a cuvette equipped with a constant stirring device and temperature controller, the system was continuously monitored at 660 nm by an UV-visible spectrophotometer.

### 4.5. Cascade Enzymatic Activity Evaluations of GOD/CAT@ZPF-Lips

GOD/CAT@ZPF-Lips cascade reaction was monitored by measuring the amount of glucose consumed, which was detected by the 3,5-dinitrosalicylic acid (DNS) method. In brief, 100 *μ*L of glucose solution (50 mM) was added to HEPES buffer (50 mM, pH = 7) dispersed with GOD/CAT@ZPF-Lips or GOD+CAT (Zn^2+^: 20 *μ*g mL^−1^) at certain time intervals (0, 1, 2, 4, 8, and 10 min), 50 *μ*L of the supernate was drawn out and mixed with 50 *μ*L DNS solution. Then, the mixtures were put in a water bath at 100°C for 5 min, after cooling to room temperature, and 400 *μ*L DI water was added. The absorbance at 560 nm was detected to monitor the concentrations of residual glucose.

### 4.6. Detection of Singlet Oxygen (^1^O_2_) In Vitro

To evaluate the generation of ^1^O_2_, H_2_O_2_, ZPF-Lips (Zn^2+^: 20 *μ*g mL^−1^, H_2_O_2_: 100 *μ*M), or GOD/CAT@ZPF-Lips (Zn^2+^: 20 *μ*g mL^−1^, H_2_O_2_: 100 *μ*M) were suspended in PBS (pH = 7.4), followed by adding DPBF (40 *μ*L, 8 mM). Then, the absorbance intensity of DPBF at 410 nm was detected by a UV-Vis spectroscope upon exposure to the US irradiation (1.0 MHz, 1.5 W/cm2, 50% duty cycle) every 1 min in dark. DPBF fade rate was calculated by the following method: *η* = *A*/*A*_0_ × 100 (where *A*_0_ represents the initial fluorescence absorption value, *A* represents the detected fluorescence absorption value). In the quantitative analysis of ^1^O_2_ based on H_2_O_2_, ZPF-Lips (20 *μ*g mL^−1^, H_2_O_2_ 100 *μ*M) or GOD/CAT@ZPF-Lips (Zn^2+^: 20 *μ*g mL^−1^, H_2_O_2_: 100 *μ*M) system was also measured by an ESR spectrometer. Furthermore, the intracellular ^1^O_2_ level was also measured using SOSG. SOSG is a fluorescence probe of ^1^O_2_, which can emit bright green fluorescence after oxidized by ^1^O_2_ facilitating the determination of the intracellular singlet oxygen levels. To test the generation of ^1^O_2_ in a normoxic environment, cancer cells were treated with ZPF-Lips (Zn^2+^: 20 *μ*g mL^−1^), ZPF-Lips (Zn^2+^: 20 *μ*g mL^−1^), or GOD/CAT@ZPF-Lips (Zn^2+^: 20 *μ*g mL^−1^) at 37°C, 5% CO_2_ under air condition for 6 h. To test the production of ^1^O_2_ in a hypoxic environment, the cells were treated with ZPF-Lips (Zn^2+^: 20 *μ*g mL^−1^) under N_2_ condition for 6 h. Then, SOSG (10 *μ*M) mixed with H_2_O_2_ (1 mL, 1 × 10^−4^ M) was added and incubated for 30 min in the air or N_2_ environment after the US irradiation (1.0 MHz, 1.5 W/cm2, 50% duty cycle) after washing the cells with PBS. Then, the excess SOSG in cells was removed by washing twice with PBS. Finally, solutions were irradiated with the US irradiation (1.0 MHz, 1.5 W/cm2, 50% duty cycle) for 5 min and fluorescence images were obtained under an CLSM (E_X_/E_M_480/535 nm).

### 4.7. In Vitro Antitumor Activity

4T1 cells were seeded in 96-well plates (1 × 10^5^ cells per well) and cultured for 12 h. In the ZPF-Lips, GOD@ZPF-Lips, and GOD/CAT@ZPF-Lips groups, the cells were treated with 100 *μ*L DMEM containing different equal concentrations of ZPF-Lips. To evaluate the influence of hypoxia in the action of sonodynamic therapy, 4T1 cells were firstly incubated with ZPF-Lips in 100.0 *μ*L of complete DMEM under hypoxia conditions or incubated with GOD@ZPF-Lips and GOD/CAT@ZPF-Lips under normal conditions for 12 h. Subsequently, the cultures were irradiated by US (1.0 MHz, 1.5 W cm^−2^, 50% duty cycle, 1 min) and then cultured for another 12 h. After incubation, the relative cell viabilities were detected by a standard CCK-8 assay. The feeding concentration in all groups was quantified by Zn^2+^: 0, 0.3, 0.6, 1.2, 2.5, 5,10, and 20 *μ*g mL^−1^.

For CLSM live/death observations, 4T1 cells (1 × 10^5^ cells) were planted on a CLSM-exclusive culture disk (*φ* = 15 mm, Corning Inc., NY, USA), and cultured for 12 h to facilitate adherence of cells. Then, 4T1 cells were treated for 24 h by the following groups: Control, US, ZPF-Lips (Zn^2+^: 10 *μ*g mL^−1^), GOD/CAT@ZPF-Lips (Zn^2+^: 10 *μ*g mL^−1^), ZPF-Lips+US (hypoxia, Zn^2+^: 10 *μ*g mL^−1^), ZPF-Lips+US (Zn^2+^: 10 *μ*g mL^−1^), GOD@ZPF-Lips+US (Zn^2+^: 10 *μ*g mL^−1^), and GOD/CAT@ZPF-Lips+US (Zn^2+^: 10 *μ*g mL^−1^). And then, cells were stained by Calcein-AM/PI, followed by observation using CLSM. In the flow cytometry analysis, 4T1 cells were dispersed in six-well microplates (1 × 10^5^ cells/plate) and treated for 24 h by ZPF-Lips (Zn^2+^: 10 *μ*g mL^−1^), GOD/CAT@ZPF-Lips (Zn^2+^: 10 *μ*g mL^−1^), ZPF-Lips+US (Zn^2+^: 10 *μ*g mL^−1^), and GOD/CAT@ZPF-Lips+US (Zn^2+^: 10 *μ*g mL^−1^). Prior to analysis, cells were harvested using trypsin and resuspended in Annexin-binding buffer (100 *μ*L) after washed twice with PBS. Then, FITC (5 *μ*L) and PI (5 *μ*L) were added to the buffer staining for 0.5 h in the dark. Finally, stained cells were analyzed by a BD LSRFortessa flow cytometer (Becton, Dickinson and Company, USA).

### 4.8. In Vivo Toxicity Study

The Kunming mice (~18 g, *n* = 5) were intravenously injected with pure saline, and saline containing GOD/CAT@ZPF-Lips (100 *μ*L, 10, 20 mg kg-1) on the first and 15th day. The body weights of mice were recorded every two days. Blood of mice was sampled in 30 days after administration for hematological and biomedical index analysis. And, their major organs (heart, liver, spleen, lung, and kidney) were harvested for hematoxylin and eosin (H&E) staining assay.

### 4.9. In Vivo Anticancer Effect Evaluation

In this experiment, tumors were planted by subcutaneously injecting 4T1 cells (1 × 10^7^ cells suspended in PBS) into the rear leg of BALB/c nude mice. After tumor volumes grew to around 50 mm^3^, mice were divided into 6 groups randomly (*n* = 5) including the following: (1) pure saline, (2) pure saline+US, (3) ZPF-Lips, (4) ZPF-Lips+US, (5) GOD/CAT@ZPF-Lips, and (6) GOD/CAT@ZPF-Lips+US. Different groups were then intravenously injected via tail veins into mice at the same doses of ZPF (20 mg kg^−1^) on 0 and 7th day. US irradiations in the above groups were performed in 6 and 12 h postinjection. Body weights and tumor volumes of mice were monitored every day after the administrations. At the end of therapy, mice were killed and their tumors were excised, weighed, and photographed. In addition, the pathological tissue sections of tumors were collected in 24 h posttreatment for H&E TUNEL and Ki-67 staining assay.

### 4.10. Statistical Analysis

Quantitative data are presented as mean ± standard deviation (s.d.). Unpaired Student's two-sided *t*-test was applied to evaluate the data by the Origin 8 software. Asterisks present significant differences (^∗^*P* < 0.1, ^∗∗^*P* < 0.05, ^∗∗∗^*P* < 0.01).

## Data Availability

All data are available from the authors upon reasonable request.

## Supplementary Materials

Supplementary Materials Fluorescent labeling and immobilizing efficiency measurement of GOD and CAT. Figure S1: CLSM images of 4T1 cells after treated under different conditions for 2 h and followed by staining with Amplex Red. Figure S2: CLSM images of 4T1 cells after incubation with FITC and RB labeled GOD/CAT@ZPF-Lips for 0.5 h (scale bar: 60 *μ*m). Figure S3: the GO enrichment histogram of the differentially expressed genes, including Molecular Function (MF), Cellular Component (CC), and Biological Process (BP). Figure S4: two significant signal transduction pathways annotated to (a) oxidative phosphorylation and (b) transcriptional misregulation in cancer. Figure S5: real-time fluorescence images of mice bearing tumor of different sizes before and after the intravenous injections of IR783 labeled ZPF-Lips. Figure S6: histological sections (H&E stained) of main organs obtained from Kunming mice sacrificed on the 30th day after injecting different doses of GOD/CAT@ZPF-Lips (0, 10, 20 mg/kg). scale bar: 150 *μ*m. Figure S7: (a) Digital photographs of excised tumors in 20 days of different treatments. (b) The body weight of 4T1 tumor-bearing BALB/c mice. Figure S8: histological sections (H&E stained) of main organs and tumors obtained from subcutaneous 4T1 tumor-bearing BALB/c mice. Table S1: zeta potentials of GOD/CAT@ZPF, Lips, and GOD/CAT@ZPF-Lips.
