# Supplementary material for: Starvation-Sensitized and Oxygenation-Promoted Tumor Sonodynamic Therapy by a Cascade Enzymatic Approach
Source: Research (Wash D C). 2021 Jun 2;2021:9769867. doi: 10.34133/2021/9769867 (PMC8214509; doi:10.34133/2021/9769867)
Supplement: Supplementary Materials — Fluorescent labeling and immobilizing efficiency measurement of GOD and CAT. Figure S1: CLSM images of 4T1 cells after treated under different conditions for 2 h and followed by staining with Amplex Red. Figure S2: CLSM images of 4T1 cells after incubation with FITC and RB labeled GOD/CAT@ZPF-Lips for 0.5 h (scale bar: 60 μm). Figure S3: the GO enrichment histogram of the differentially expressed genes, including Molecular Function (MF), Cellular Component (CC), and Biological Process (BP). Figure S4: two significant signal transduction pathways annotated to (a) oxidative phosphorylation and (b) transcriptional misregulation in cancer. Figure S5: real-time fluorescence images of mice bearing tumor of different sizes before and after the intravenous injections of IR783 labeled ZPF-Lips. Figure S6: histological sections (H&E stained) of main organs obtained from Kunming mice sacrificed on the 30th day after injecting different doses of GOD/CAT@ZPF-Lips (0, 10, 20 mg/kg). scale bar: 150 μm. Figure S7: (a) Digital photographs of excised tumors in 20 days of different treatments. (b) The body weight of 4T1 tumor-bearing BALB/c mice. Figure S8: histological sections (H&E stained) of main organs and tumors obtained from subcutaneous 4T1 tumor-bearing BALB/c mice. Table S1: zeta potentials of GOD/CAT@ZPF, Lips, and GOD/CAT@ZPF-Lips. [file 9769867.f1.docx]

**Starvation-****Sensitized and Oxygenation-Promoted Tumor Sonodynamic Therapy by A Cascade Enzymatic Approach**

Wencheng Wu^1, 2^, Yinying Pu^3^, Han Lin,^1^ Heliang Yao,^1^and Jianlin Shi^1, 2,4*^

^1^The State Key Lab of High Performance Ceramics and Superfine Microstructures, Shanghai Institute of Ceramics, Chinese Academy of Sciences, Shanghai 200050, P. R. China. E-mail: [jlshi@mail.sic.ac.cn](mailto:jlshi@mail.sic.ac.cn)

^2^Center of Materials Science and Optoelectronics Engineering, University of Chinese Academy of Sciences, Beijing 100049, P. R. China.

^3^Department of Medical Ultrasound, Shanghai Tenth People’s Hospital, Ultrasound Research and Education Institute, Tongji University School of Medicine, Shanghai 200072, P. R. China.

^4^Platform of Nanomedicine Translation, Shanghai Tenth People’s Hospital of Tongji University, Shanghai 200072, P. R. China.

***Supplementary experiments***

**Fluorescent labeling and** **immobilizing efficiency measurement of GOD and CAT.** For the labeling of GOD by FITC, firstly, the pH of the GOD solution (5 mL, 2 mg mL^-1^) was titrated to 9.5 with sodium carbonate buffer. Then, the FITC (1 mg) was added and stired for 3 h. They were linked together by the reaction between isothiol acid groups and free amino groups of GOD. Similar method was used to complete the marking of Rhodamine b (RB) to CAT. For measuring immbolized efficiency of GOD and CAT at different feeding amounts, zinc nitrate aqueous solution (2 mL59.7 mg mL^-1^) was added into to a DI water solution (4 mL) containing 2-hydroxy-5- fluoropyrimidine (138.4 mg). Then, FITC labeled GOD (5 mg) and RB labeled CAT (10 mg) were added. The reaction mixture was stirred for 20 minutes at 25 ℃. The product was centrifugated and their supernatant were colleted. The fluorescence intensity of FITC and RB in the initial solution and the supernatant was measured by UV-visible spectroscopy. The immbolization efficiency data were measured according to the method: η = A/A_0_ × 100 (where A_0_ represents the initial fluorescence absorption value, A represents the detected fluorescence absorption value). The concentrations of GOD and CAT were evaluated against a calibration curve, which was established by recording the UV-Vis spectra and the absorbance of varied known concentrations of GOD or CAT.

**Detection of O_2_ in vitro.** RDPP, an oxygen indicator, was used to decte O_2_ production based on its proportional fluorescence intensity decrease at increased oxygen concentration. Aqueous solutions containing H_2_O_2_, H_2_O_2_ + ZPF-Lips (Zn^2+^: 20 μg mL^−1^, H_2_O_2_: 100 μM, a pathophysiological concentration identified in the native tumor), or H_2_O_2_ + GOD/CAT@ZPF-Lips (Zn^2+^: 20 μg mL^−1^, H_2_O_2_ : 100 μM) were added into separate tubes. The concentration of H_2_O_2_ was 1 × 10^-4^ M. Then, 0.2 mL of liquid paraffin was added on top of each tested solution to isolate it from the air. The fluorescence intensity of RDPP (λex = 560 nm) at 645 nm was recorded at different time points. The fluorescence intensity decay of RDPP by O_2_ was caculated by this method: η = A/A_0_ × 100 (where A_0_ represents the initial fluorescence absorption value, A represents the detected fluorescence absorption value). The intracellular generation of O_2_ was also explored using RDPP, in which the red fluorescence intensity of RDPP increases proportionally with the increases of hypoxic level. Briefly, 4T1 cells were incubated with ZPF-Lips, GOD@ZPF-Lips, and GOD/CAT@ZPF-Lips (Zn^2+^: 20 μg mL^−1^) for 6 h in the N_2_ atmosphere with additionanl H_2_O_2_ (10^-4^ M). Upon discarding the medium, cells were rinsed carefully with PBS and incubated with RDPP probe for 30 min. The cells were then observed under CLSM.

***In vitro* cellular-uptake.** 4T1 cells were seeded in confocal dishes (2×10^5^ cells per well) for 12 h. Then, the cells were incubated with free FITC and RB labeled GOD/CAT@ZPF-Lips (Zn^2+^: 20 μg mL^−1^) for 4 h. The residual materials in cell dishs were rmoved by washing with PBS, then, the cellular uptake was observed by CLSM.

**Detection of H_2_O_2_ in vitro.** H_2_O_2_ detection experiment was carried out by adding GOD@ZPF-Lips or free GOD at the equal GOD concentration of 20 μg mL^-1^ into glucose (5 mM) solutions. In different time intervals, the mixtures were centrifuged and the generation of H_2_O_2_ was recorded by UV-Vis spectra. During the intracellular detection, first, 1.0 × 10^5^ cells were seeded on confocal dishes for 12 h. Then cells were treated with ZPF-Lips, GOD@ZPF-Lips, and GOD/CAT@ZPF-Lips (Zn^2+^: 20 μg mL^−1^) for 4 h. After washed for three times with PBS, the cells in different groups were incubated with a fluorescent probe (Amplex Red) for 15 min. Finally, the residual fluorescent probe in cells were removed by PBS and imaged under a confocal laser scaning microscope (CLSM).

**Glucose uptake.** The cellular glucose uptake was measured using 2-NBDG, a fluorescent glucose analogue. Briefly, 4T1 cells were seeded in a CLSM-exclusive culture disk (φ = 15 mm, Corning Inc., NY, USA), and incubated for 12 h to facilitate the adherence of cells. Then, 4T1 cells were treated with free DMEM and DMEM containing GOD@ZPF-Lips (Zn^2+^: 5, 10 μg mL^−1^) for 4 h. After washed with PBS twice, 2-NBDG (10 μM) were added and cultured for another 30 min. Finally, the fluorescence of the samples was monitored by a BD LSRFortessa flow cytometer at an excitation wavelength of 488 nm and an emission wavelength of 540 nm.

**Cellular senescence detection.** 4T1 cells seeded in a CLSM-exclusive culture disk were treated by DMEM containing ZPF-Lips (Zn^2+^: 5 μg mL^−1^), GOD@ZPF-Lips (Zn^2+^: 5 μg mL^−1^) and GOD/CAT@ZPF-Lips (Zn^2+^: 5 μg mL^−1^) for 12 h. Cells treated with free DMEM were set as control. Then, the senescences of cells were detected by a cellular senescence detection kit-SPiDER-βGal. The specific steps were carried out according to the instructions of the manual.

**Animal research.**Female Kunming mice (8 weeks) and BALB/c nude mice (6 weeks) were purchased from Shanghai SLAC Laboratory Animal Company. All experimental protocols were approved by the Laboratory Animal Center of Shanghai Tenth Peoples’ Hospital and complied with policies of the National Ministry of Health.

***In vivo* blood circulation of GOD/CAT@ZPF-Lips.** GOD/CAT@ZPF-Lips (100 μL, 20 mg kg^-1^) were intravenously injected into mice (n = 3), and equal blood (20 μL, n = 3) was taken in different time durations (2, 5, 10, 20, and 30 min, 1, 2, 4, 8, 12, and 24 h). Then, the Zn contents in blood samples were analyzed by ICP-OES after digesting by chloroazotic acid for 24 h.

***In vivo* biodistribution.** When the 4T1 tumor volume (n = 3) grew to around 200 mm^3^, saline solution containing GOD/CAT@ZPF-Lips (100 μL, 20 mg kg^-1^) was administered intravenously. The mice were sacrified in 4, 12, and 24 h post injection. Their main organs (heart, liver, lung, spleen, kidney) and tumors were harvested. The Zn concentrations in organs and tumors were measured by ICP-OES after digesting by chloroazotic acid for 24 h.

***In vivo* imaging and biodistribution analysis.** When the volumes of the 4T1 tumors grew to around 200 mm^3^, IR783 labelled GOD/CAT@ZPF-Lips was injected into the BABL/C mice intravenously (100 μL, mg kg^-1^). The fluorescence signals of IR783 were detected by an *ex*/*in vivo* imaging system (VISQUE Invivo Smart-LF, Korea). The mice were sacrificed in 24 h post-injection. Their main organs (heart, liver, spleen, lung, kidneys) and tumors were collected for semiquantitative biodistribution analysis and imaging using the *ex*/*in vivo* imaging system.

***In vivo* ROS evaluation.** 4T1 tumor-bearing mice were treated by pure saline, pure saline + US, ZPF@Lips, ZPF@Lips + US, GOD/CAT@ZPF@Lips, GOD/CAT@ZPF@Lips + US, respectively when the tumor volume reached 200 mm^3^. These mice were sacrified in 12 h post-injection, and their tumors were immediately collected for DCFH-DA staining.

***Supplementary figures***


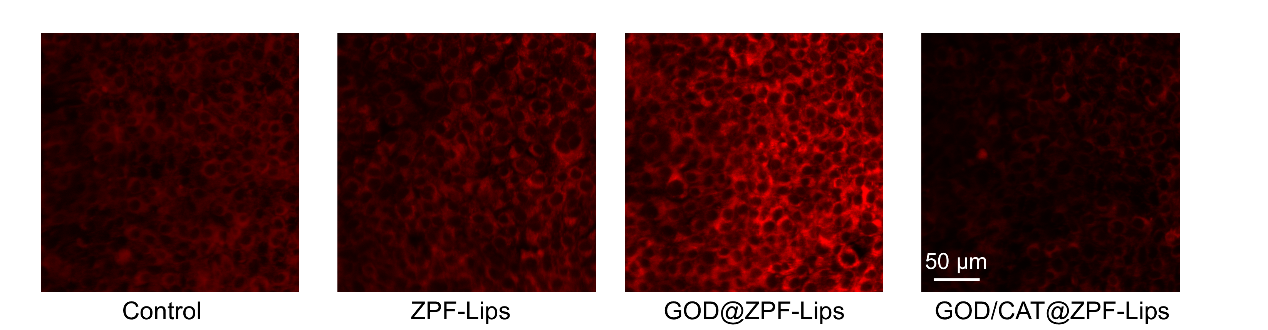


**Figure S1.** CLSM images of 4T1 cells after treated under different conditions for 2 h and followed by staining with Amplex Red.


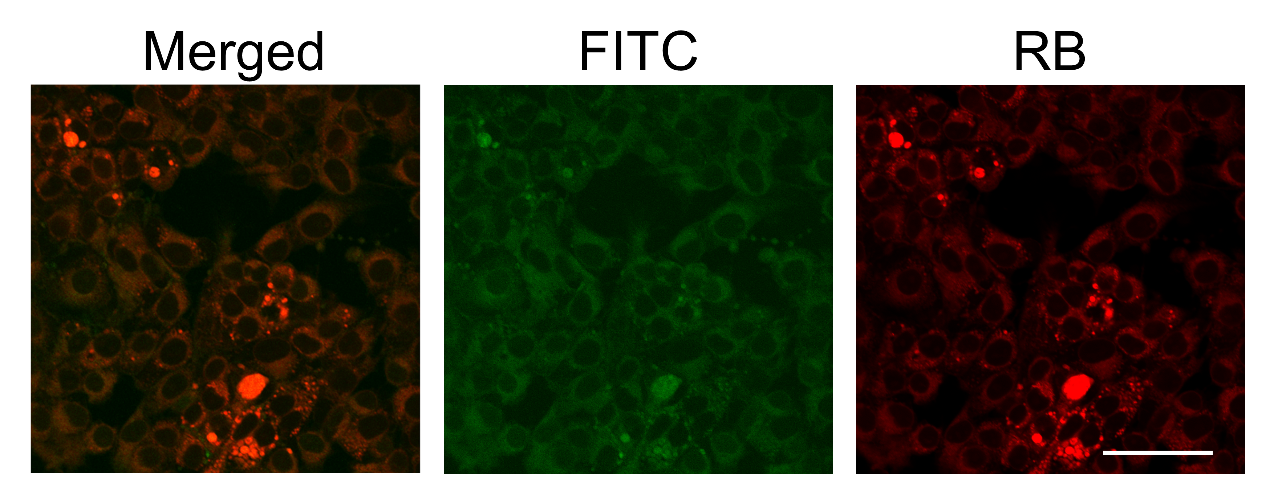


**Figure S2.** CLSM images of 4T1 cells after incubation with FITC and RB labeled GOD/CAT@ZPF-Lips for 0.5 h (scale bar: 60 μm).


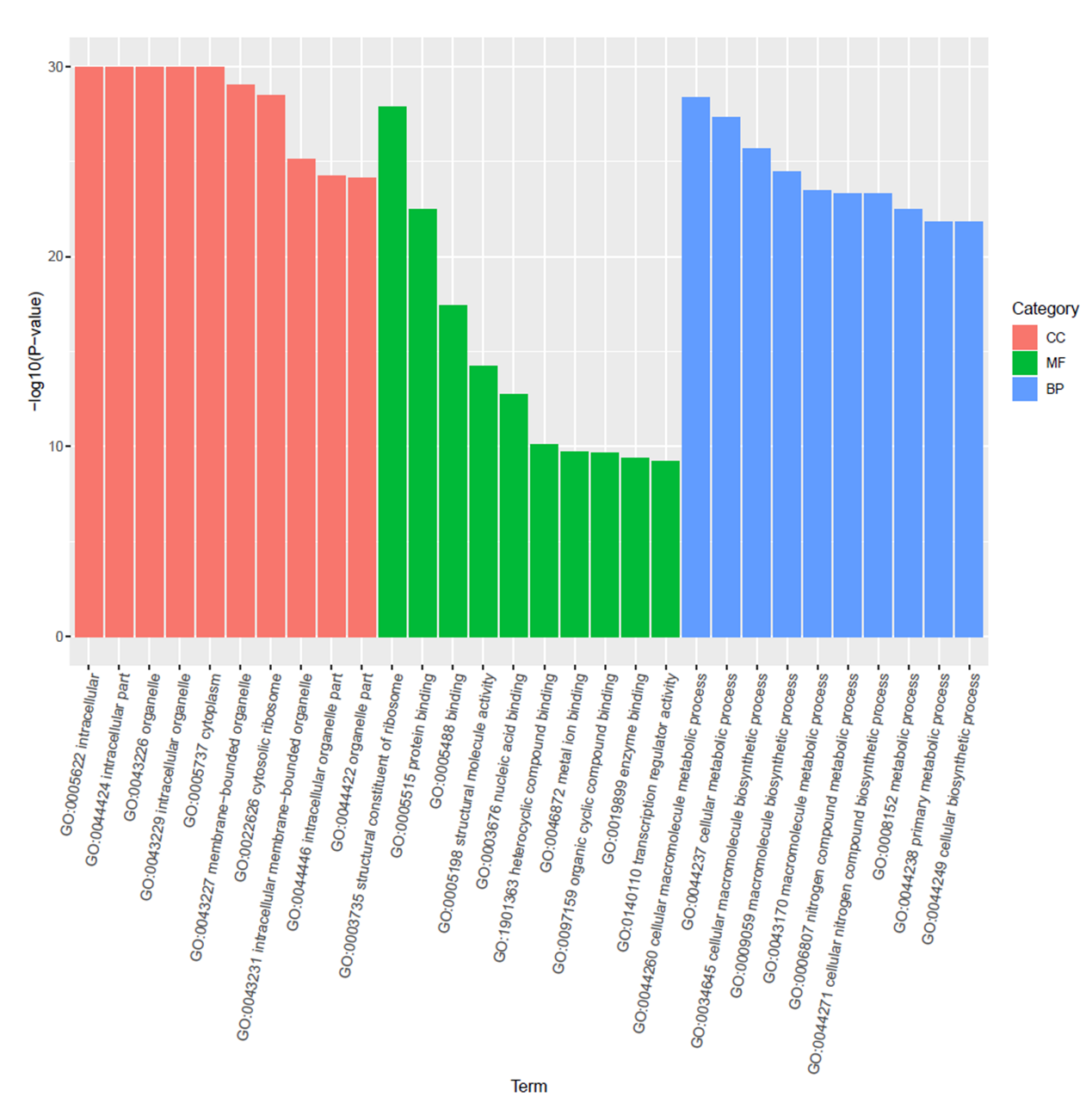


**Figure S3.** The GO enrichment histogram of the differentially expressed genes, including Molecular Function (MF), Cellular Component (CC), and Biological Process (BP).


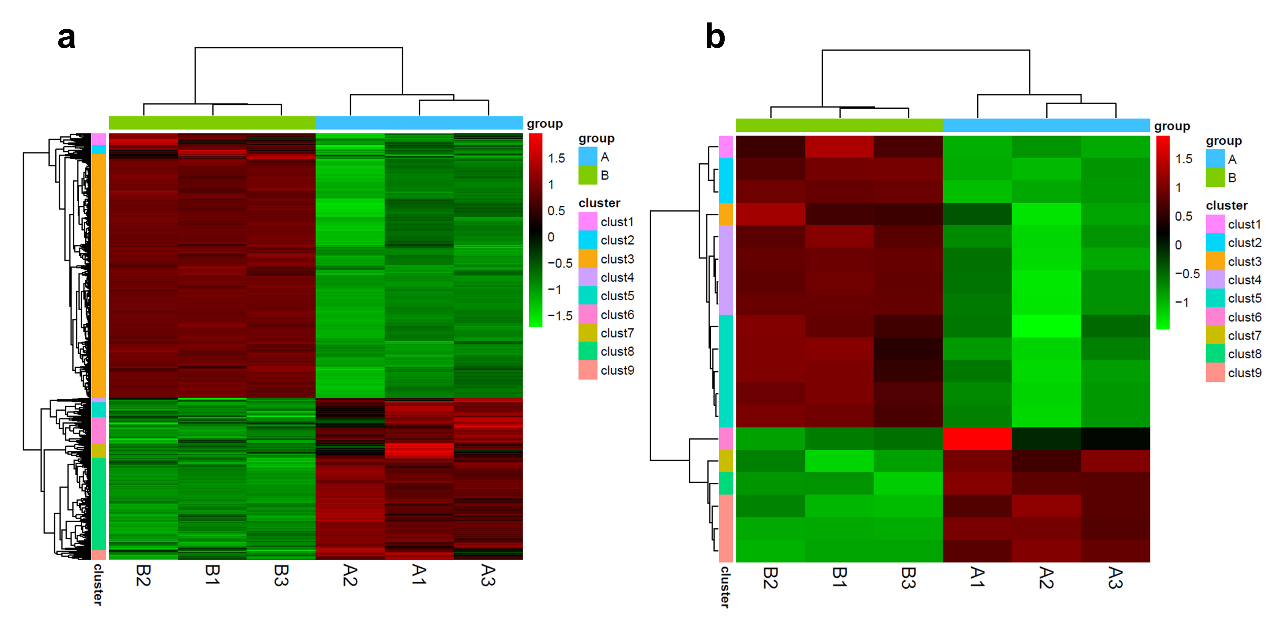


**Figure S4.** Two significant signal transduction pathways annotated to (a) oxidative phosphorylation and (b) transcriptional misregulation in cancer.


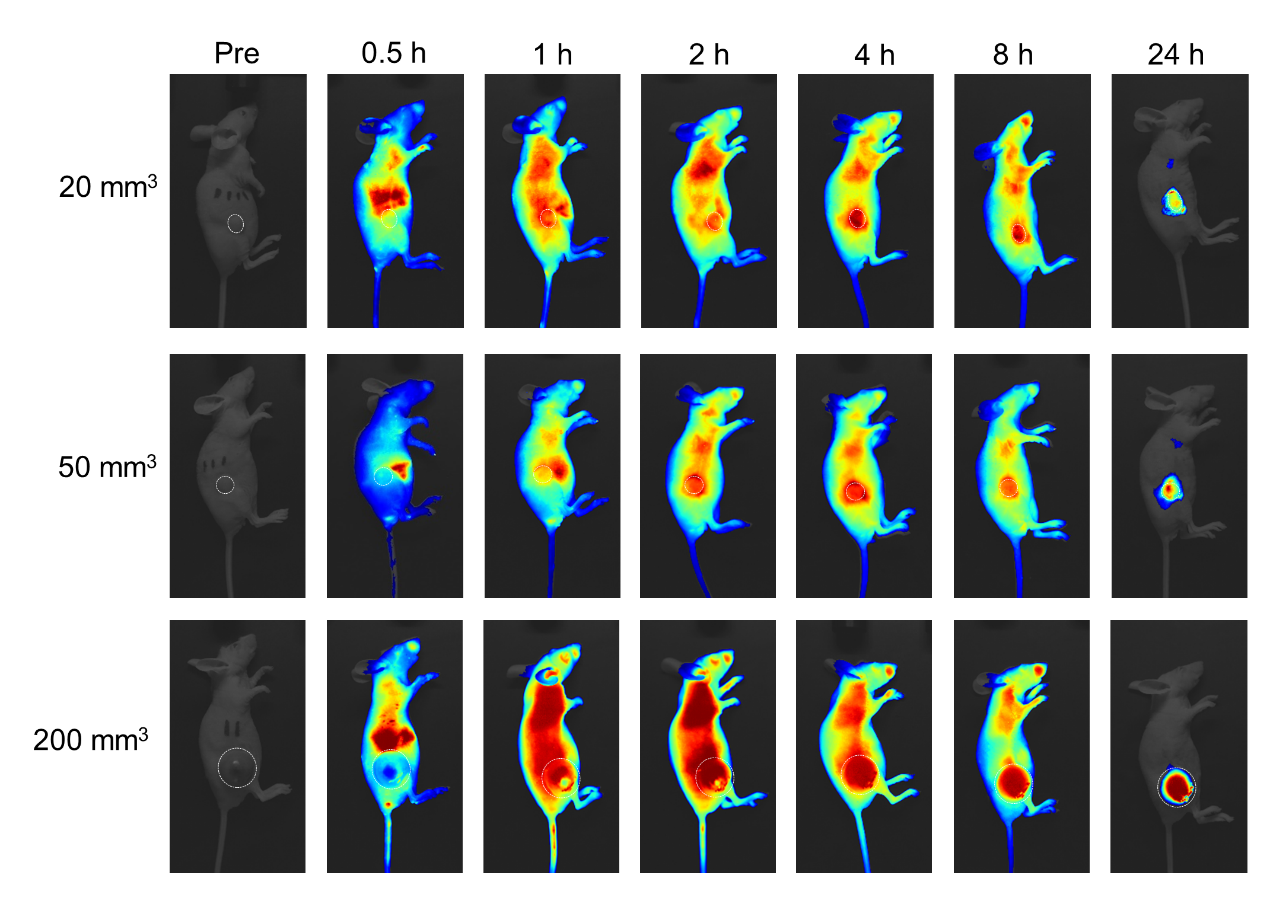


**Figure S5.** Real-time fluorescence images of mice bearing tumor of different sizes before and after the intravenous injections of IR783 labeled ZPF-Lips.


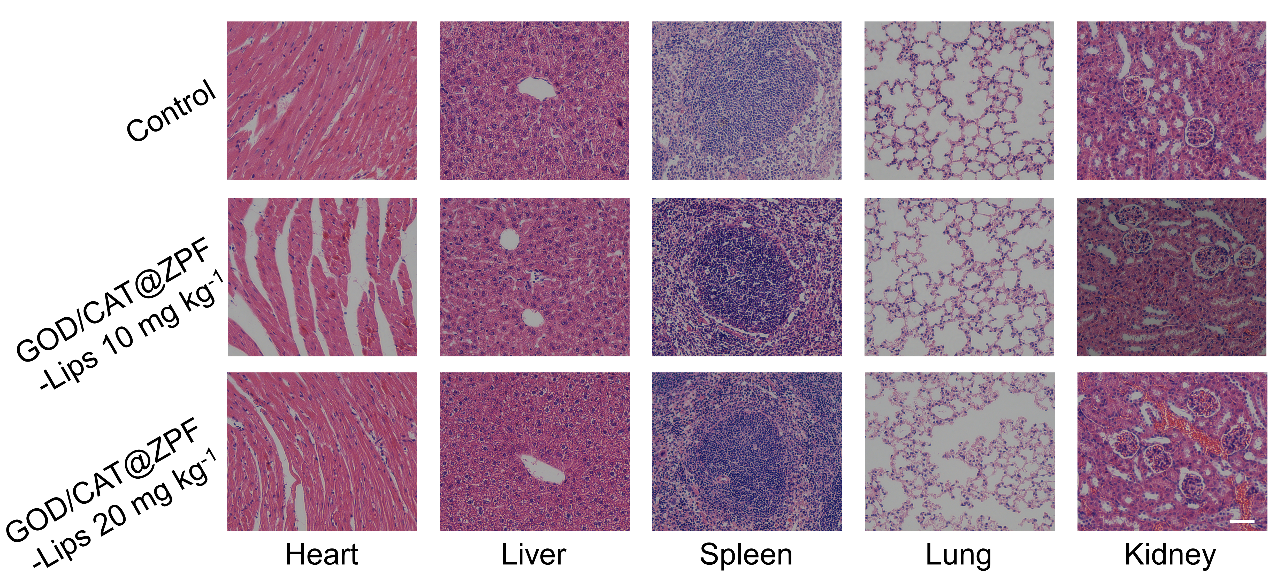


**Figure S6.** Histological sections (H&E stained) of main organs obtained from Kunming mice sacrificed on the 30^th^ day after injecting different doses of GOD/CAT@ZPF-Lips (0, 10, 20 mg/kg). scale bar: 150 μm.


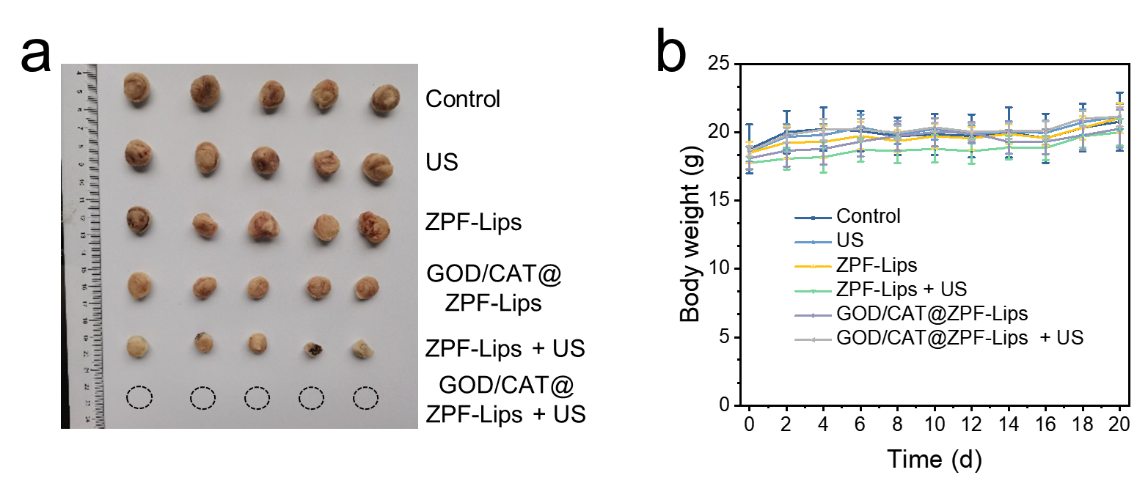


**Figure S7.** (a) Digital photographs of excised tumors in 20 days of different treatments. (b) The body weight of 4T1 tumor-bearing BALB/c mice.


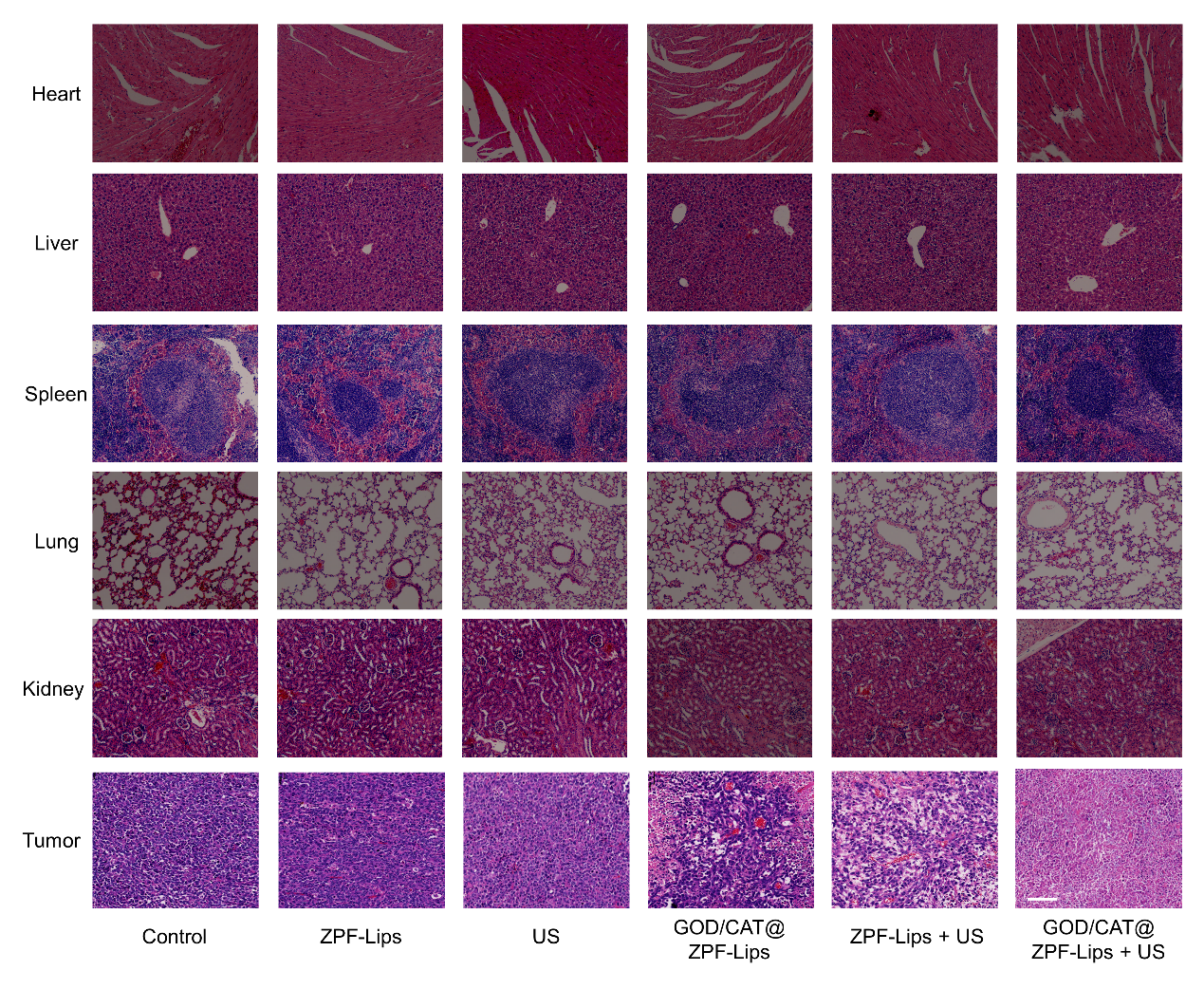


**Figure S8.** Histological sections (H&E stained) of main organs and tumors obtained from subcutaneous 4T1 tumor-bearing BALB/c mice.

**Table S1.** Zeta potentials of GOD/CAT@ZPF, Lips, and GOD/CAT@ZPF-Lips.

| GOD/CAT@ZPF | Lips | GOD/CAT@ZPF-Lips |  |
| --- | --- | --- | --- |
| 10.13 mV | -30.05 mV | -9.56 mV |  |
